# Tuberculous Meningitis Treated by Sterilized Air

**Published:** 1918-08

**Authors:** 


**Tuberculous Meningitis Treated by Sterilized Air.** Felix Ramond and Francois have treated 9 patients by withdrawing about 40 c.c. of fluid by lumbar puncture and injecting air sterilized by passage through a red hot platinum canula, sufficiently long to allow cooling to room temperature before reaching the cerebro-spinal space. They warn against the consequent change in volume if the air is injected at temperatures much different from that of the body. They admit that all the cases died but temporary improvement was noted in one. The analogy to the treatment of tuberculous peritonitis is obvious.

